# Use of myocardial perfusion imaging and estimation of associated radiation doses in Germany from 2005 to 2012

**DOI:** 10.1007/s00259-013-2683-5

**Published:** 2014-02-12

**Authors:** O. Lindner, F. M. Bengel, M. Hacker, W. Schäfer, W. Burchert, Working Group Cardiovascular Nuclear Medicine of the German Society of Nuclear Medicine

**Affiliations:** 1 Institute of Radiology, Nuclear Medicine and Molecular Imaging, Heart and Diabetes Center North Rhine-Westphalia, University Hospital of the Ruhr University Bochum, Georgstr. 11, 32545 Bad Oeynhausen, Germany; 2 Department of Nuclear Medicine, Hanover Medical School, Hanover, Germany; 3 Department of Biomedical Imaging and Image-guided Therapy, Division of Nuclear Medicine, Medical University of Vienna, Vienna, Austria; 4 Clinic of Nuclear Medicine, Kliniken Maria Hilf GmbH, Mönchengladbach, Germany

**Keywords:** Myocardial perfusion scintigraphy, Survey, Utilization statistics, Radiation exposure

## Abstract

**Purpose:**

For several years the Working Group Cardiovascular Nuclear Medicine of the German Society of Nuclear Medicine has been performing a regular survey to obtain information on technique, utilization and development of myocardial perfusion scintigraphy (MPS). Currently, data of six surveys from 2005 to 2012 are available. The aim of this paper is to deliver a general and comprehensive overview of all surveys documenting the course of patient doses over time and the development of the method.

**Methods:**

A one-page questionnaire with number of MPS patients, number of stress and rest MPS, referral structure and several technical issues was sent to all centres performing MPS in Germany and evaluated. With the data on protocol utilization, effective MPS patient doses were estimated.

**Results:**

MPS per million population (pmp) varied between 2,380 and 2,770. In 2012, MPS pmp showed a slight increase for the first time. From 2005 to 2009 the angiography to MPS ratio increased from 3.4 to 4.4, and the revascularization to MPS ratio decreased from 0.66 to 0.53. In 2012, both indices demonstrated an opposite trend for the first time (4.1 and 0.55). A total of 108 centres participated in all surveys. They showed an increase in MPS patients of 4.0 % over the reporting period. In 2012, more than 50 % of the centres experienced no change or an increase in MPS numbers. The leading single competitor was MRI, followed by angiography and stress echocardiography. ^201^Tl studies have decreased since 2005 from 20 to 5 %. ^99m^Tc MPS studies showed a mild increase in 2-day protocols. In 2012, the average effective dose per patient was estimated at 7.4 mSv. Due to the decreasing use of ^201^Tl, a mild decline over the observation period can be documented. Dynamic exercise stress was the most common stress test and adenosine the leading pharmacological stress agent, with a growing percentage. In 2012, the regadenoson percentage was 9 %. Gated single photon emission computed tomography (SPECT) noted an increasing acceptance with >70 % in 2012. The segmental scoring of perfusion studies had a low acceptance. Ambulatory care cardiologists represented the major referral group.

**Conclusion:**

Germany has a moderate to moderate-high MPS utilization rate. Nevertheless, coronary artery disease (CAD) diagnosis and disease management are dominated by angiography. The survey data reveal a positive trend in MPS and a decrease in average patient dose reflecting good practice with guideline adherence, the implementation of technical improvements and success in training.

## Introduction

Data on myocardial perfusion scintigraphy (MPS), especially long-term data about routine use and practice, are of growing interest in times of competitive methods, dose reduction approaches and quality assurance. Thus, for several years, the Working Group Cardiovascular Nuclear Medicine of the German Society of Nuclear Medicine has been performing a regular survey in order to obtain reliable information on technique, practice, utilization and development of MPS. Combining the data on protocol utilization with the German diagnostic reference levels (DRL, derived from implementation of the Euratom directive), values of effective patient doses from MPS can be estimated and monitored over time [1].

With the 2012 inquiry, data of six surveys covering the reporting years 2005–2009 and 2012 are available. The detailed annual results of private practices, general hospitals and university hospitals were published elsewhere [2–7]. The aim of this paper is to deliver a more general and comprehensive overview of all surveys documenting the development of the method and the average radiation doses, the latter being of special interest in the comparison with competitive methods and measures to reduce patients’ radiation exposure.

Long-term survey data are rare. The European Council of Nuclear Cardiology (ECNC), a joint organization of the European Association of Nuclear Medicine (EANM) and the European Society of Cardiology (ESC), which strives to promote clinical and professional activity in nuclear cardiology, published two survey reports on the years 2005 and 2007 [17, 26]. More recently, the American Society of Nuclear Cardiology (ASNC) conducted a random sample survey of members in 2011. Data on the practice of nuclear cardiology were published in 2012 [8] and on protocols and doses in 2013 [9].

Since Germany is the most populous country in Europe, the German surveys may reflect to some extent developments in daily and routine MPS practice in Europe and supplement the European report data of 2005 and 2007. The course of the patient numbers is, however, difficult to interpolate to other countries as national reimbursement regulations have great impact and may even dilute or delay trends resulting from evidence-giving studies.

## Materials and methods

### Survey and data management

Based on the register of members of the Germany Society of Nuclear Medicine, registers of office-based nuclear medicine physicians of the 17 state chambers of physicians and the German hospitals’ address book, potential facilities or physicians practising nuclear medicine were identified and updated before every survey.

A one-page questionnaire with a cover letter was sent by fax. In cases of no response a first reminder was forwarded 4 weeks later and a second 4–6 weeks after that.

All questionnaires comprised: (1) number of patients with MPS, (2) number of stress and rest MPS, (3) stress techniques, (4) radiopharmaceuticals, (5) protocols, (6) utilization of gated single photon emission computed tomography (SPECT) and (7) the referral structure. Since 2007 the frequency of a quantitative perfusion analysis has been requested. In 2006, 2007 and 2012 data on change of MPS utilization, in 2006 and 2009 data on attenuation correction (AC), in 2006 data on indications [detection of coronary artery disease (CAD)], in 2007 data on diabetics and in 2008 data on age structure were additionally surveyed.

In order to verify the representativeness of the survey and to estimate the total or “true” MPS numbers reliably, the survey results were related to the data of the National Association of Statutory Health Insurance Physicians (NASHIP) [Kassenärztliche Bundesvereinigung (KBV)] [10]. The NASHIP data represent the official number of ambulatory statutory health insurance patients.

The estimate of the total MPS numbers was based on the fact that three groups of MPS patients were not covered by the NASHIP statistics: (1) privately insured patients, (2) inpatients of hospitals and (3) hospital patients of private practices. Data concerning the last two items were derived from the surveys. MPS numbers from private insurance companies were not available and estimated from the percentage of privately insured patients documented in the 2007 and 2011 German microcensus. The percentage amounted to 11.8 % in 2007 and to 12.8 % in 2011 (mean 12.4 %) [11, 12]. Based on these data, a proportion of 12.4 % MPS of privately insured patients was taken for the missing data concerning item 1 to determine the true annual MPS numbers.

Accordingly, 50–55 % of all MPS were evaluated with every query and the true annual MPS patient numbers estimated to be twice as high as the survey patient numbers, which were therefore multiplied by 2. This approach results in a slight overestimation of the total MPS number by < 10 %.

For utilization calculation the true number of annual MPS patients was divided by the current population statistics. The results were related to the angiography numbers of the annual German report on cardiology [13]. Revascularization case numbers were also taken from the annual cardiology statistics. At the time of writing this paper, only cardiology data up to 2011 were available, and they related to the MPS 2012 numbers.

### Estimated effective doses

The radiopharmaceutical activities used in the different protocols for effective dose calculation are listed in Table 5. They are derived from DRL for the most frequent nuclear medicine studies. DRL were designated by the German Federal Office for Radiation Protection in 2003, implementing the Council Directive 97/43 Euratom [14, 15]. DLR represent the *average* radiopharmaceutical activities. Accordingly, in a 2-day protocol 2 × 600 MBq can be applied, whereas in a 1-day protocol the first injection should amount to 250 MBq and the second to 750 MBq. Thus, it is also appropriate and dose saving to inject 2 × 250 MBq in a 2-day protocol. Additionally, multi-head SPECT cameras deliver 250 MBq scans with an excellent image quality. In light of these considerations, lower and upper activity ranges for the average effective dose calculations of the different protocols were defined (Table 5). For ^201^Tl an effective dose of 0.22 mSv/MBq was taken and for ^99m^Tc radiopharmaceuticals at stress of 0.008 mSv/MBq and at rest of 0.009 mSv/MBq [16]. The last two refer to ^99m^Tc-sestamibi. The effective dose of ^99m^Tc-tetrofosmin is slightly lower but was equated with ^99m^Tc-sestamibi.

### Statistical analysis

Data are presented as numbers and percentage of observation when appropriate. Statistical analyses for significant changes in the course of the surveys were performed using the Kruskal-Wallis test and the chi-square test when appropriate. For the analyses the statistical software package IBM SPSS, version 20 (IBM Corp., Armonk, NY, USA) was used. A *p* value <0.05 was considered statistically significant.

## Results

### MPS patient figures and utilization data from cardiology

Table 1 lists the MPS patient numbers of the particular survey, the estimated total patient numbers after adjustment to the NASHIP statistics and MPS per million population (pmp). During the period under review, mean and median MPS figures mildly increased but differ, which accounts for a large percentage of centres with only a few studies. Since 2006 a mild decrease in centres with <50 MPS/year (<1 MPS/week) has been observed and, on the other hand, a mild increase in centres with >1,000 MPS/(>4 MPS/day) since 2007. For the first time, MPS studies pmp showed a slight increase from 2009 to 2012 and currently reach the 2007/2008 level.

**Table 1:** MPS utilization and local activity

	2005	2006	2007	2008	2009	2012
MPS patients (survey)	112,136	108,939	114,374	99,879	98,253	105,941
Total MPS patients^a^	224,272	217,878	228,748	199,758	196,506	211,882
MPS pmp	2,720	2,640	2,770	2,420	2,380	2,580
No. of centres	356	353	370	312	292	278
Mean MPS	315	309	309	320	336	381
Median MPS	160	144	141	147	163	179
< 50 MPS/year	18.5 %	28.6 %	25.4 %	26.9 %	23.3 %	20.9 %
> 1,000 MPS/year	7.6 %	8.5 %	7.6 %	8.7 %	8.6 %	10.4 %

^a^The adjustment by the NASHIP (National Association of Statutory Health Insurance Physicians) statistics revealed that about 50–55 % of all MPS were evaluated in every query. To obtain the total MPS patient number the survey numbers were multiplied by 2

**Fig. 1:**
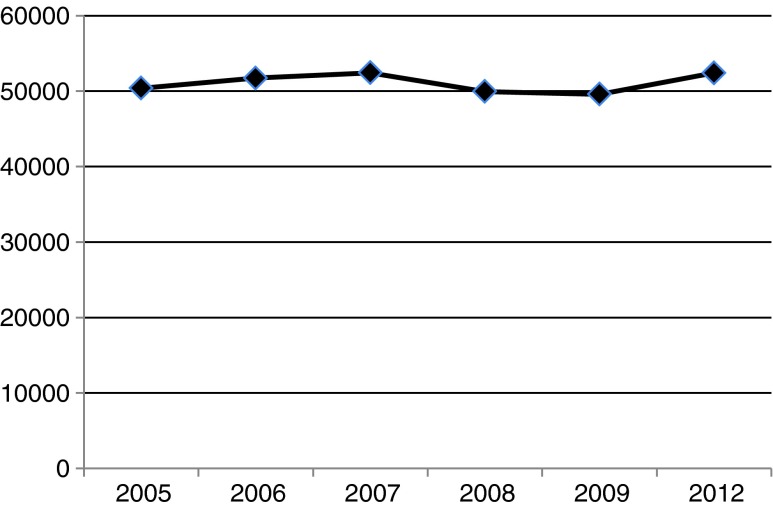
Numbers of MPS patients of the 108 centres participating in all surveys from 2005 to 2012

A total of 108 centres delivered data in all six surveys. They demonstrate an increase in MPS patients of 4.0 % (50,392 to 52,400) from 2005 to 2012 (Fig. 1).

The number of angiographies, interventions and ratios are presented in Table 2. In 2011, the angiography numbers slightly declined for the first time since 1980, by 1.3 %. Bypass surgery figures decreased and percutaneous transluminal coronary angioplasty (PTCA) figures increased over the observation period. The total revascularization figures increased and the angiography to revascularization ratio remained constant. From 2005 to 2009, the angiography to MPS ratio increased and the revascularization to MPS ratio decreased. In 2012, both demonstrated opposite trends for the first time.

**Table 2:** Angiographies, interventions and ratios

	2005	2006	2007	2008	2009	2010	2011	2012
Angiography	772,137	806,533	832,420	851,517	864,858	881,514	870,282	^a^
Angiography pmp	9,370	9,800	10,130	10,380	10,400	10,690	10,630	^a^
Angiography/MPS	3.4	3.7	3.6	4.3	4.4	–	–	4.1^b^
Revascularization (total)	338,300	355,552	363,054	365,777	368,661	381,865	383,953	^a^
PTCA	270,964	291,050	299,690	304,719	310,166	325,872	328,654	^a^
Bypass	67,336	64,502	63,364	61,058	58,495	55,993	55,299	^a^
Angiography/revascularization	2.3	2.3	2.3	2.3	2.3	2.3	2.3	^a^
Revascularization/MPS	0.66	0.61	0.63	0.55	0.53	–	–	0.55^b^

^a^Data still unpublished at the time of writing

^b^The MPS 2012 numbers were related to cardiology data from 2011

### Individual changes in referral behaviour and competitive methods

In 2006, 2007 and 2012 the participants were asked to assess their individual development of MPS numbers and changes by competitive methods. Their estimates are given in Table 3. In 2012 more than 50 % of the centres experienced no change or even an increase in MPS numbers. The percentage of those with decreasing MPS numbers was lowest in 2012. The leading single competitor was MRI, followed by angiography and stress echocardiography. CT still seemed to play a minor role. The greatest competition, however, was not experienced by one single but by several competitive imaging methods.

**Table 3:** Per cent of survey participants with changes in referral behaviour by competitive methods

	2006	2007	2012
Unknown	31 %	32 %	22 %
No change	45 %	40 %	29 %
More	^a^	^a^	27 %
Less	24 %	28 %	21 %
Less by			
Cardiac MRI	30 %	23 %	22 %
Stress echo	18 %	16 %	12 %
Angiography	8 %	11 %	15 %
Cardiac CT	6 %	7 %	3 %
More than 1 method	38 %	43 %	32 %
Unknown	^a^	^a^	16 %

Percentages do not always total 100 because of rounding

^a^Not questioned

### Protocols, radiopharmaceuticals and effective doses

The utilization frequency of typical study protocols is given in Table 4 (upper half). Hybrid protocols were only questioned in 2005 and thereafter omitted due to their very low proportion. In 2005, no splitting of ^99m^Tc protocols into 1-day or 2-day protocols occurred, and in all surveys there was no differentiation of ^99m^Tc radiopharmaceuticals (sestamibi or tetrofosmin).

**Table 4:** Utilization of protocols and average effective patient doses

		2005	2006	2007	2008	2009	2012	*p* value
Protocols	^201^Tl	20 %	15 %	17 %	12 %	11 %	5 %	<0.001
	^99m^Tc stress only	15 %	14 %	12 %	15 %	13 %	16 %	0.006
	^99m^Tc 2-day	61 %	47 %	46 %	45 %	47 %	52 %	<0.001
	^99m^Tc 1-day		22 %	23 %	26 %	28 %	25 %	0.03
	^99m^Tc rest	2 %	2 %	2 %	2 %	2 %	2 %	n.s.
	^99m^Tc, ^201^Tl (hybrid)	2 %	–	–	–	–	–	–
Average effective doses	Average max. patient dose (mSv)	^a^	9.8	10.2	9.7	9.7	9.2	<0.001
	Mean patient dose (mSv)	^a^	8.2	8.7	8.1	8.1	7.4	<0.001
	Average min. patient dose (mSv)	^a^	6.6	7.1	6.5	6.5	5.6	<0.001

Percentages do not always total 100 because of rounding

^a^No calculation of doses; 1-day and 2-day protocols were not questioned

^201^Tl studies have been decreasing continuously since 2005. Their proportion reached only 5 % in 2012. ^99m^Tc MPS studies showed a mild increase in 2-day protocols. The proportions of the other ^99m^Tc protocols varied to some extent over time, but remained on the same level.

**Fig. 2:**
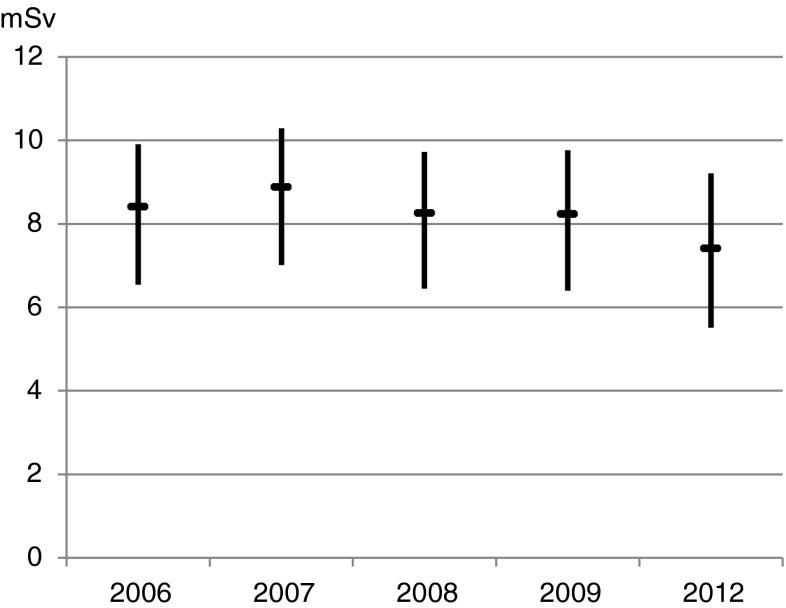
Average, maximal and minimal patient dose per MPS based on lower and upper levels of administered activities (Table 5) and protocol use from 2006 to 2012

Table 5 lists the administered radiopharmaceutical activities and Table 4 (lower half) the average effective dose range of an MPS patient according to the utilization of the protocols. The data document an average effective dose per patient of 7.4 mSv in 2012 and, due to the decreasing use of ^201^Tl, a mild decline over the observation period of 7 % for the average upper and 16 % for the average lower patient doses (Fig. 2).

**Table 5:** Protocols, administered activity ranges and effective doses

Radiopharmaceutical	Protocol	DRL^a^	Administered activity range		Effective dose range
			Average lower level	Average upper level	
		(MBq)	(MBq)	(MBq)	(mSv)
^201^Tl		75	75	75	16.5
^99m^Tc-sestamibi or ^99m^Tc-tetrofosmin	Stress only	600	250	600	2.0–4.8
	Rest only	600	250	600	2.3–5.4
	1-day protocol	1,000	250 stress, 750 rest*	250 stress, 750 rest*	8.8
	2-day protocol	1,200	250 stress, 250 rest	600 stress, 600 rest	4.3–10.2

*According to DRL no definition of lower and upper dosage levels

^a^Diagnostic reference level of average radiopharmaceutical activities according to [14, 15]

### Stress techniques

Table 6 shows the utilization of stress techniques. Dynamic exercise stress was the most common stress test, but its share continuously decreased. Adenosine represented the leading pharmacological stress agent with a growing percentage. Dipyridamole is not licensed as a MPS stressor in Germany and can be applied only under certain circumstances. Thus, its acceptance has decreased. Regadenoson was licensed in Germany in 2011 and has been available since September 2011. Its share reached 8 % in 2012. All vasodilator stress tests were increasingly combined with low-level exercise. Dobutamine was only used to a very low extent.

**Table 6:** Stress techniques in MPS

	2005	2006	2007	2008	2009	2012	*p* value
Ergometry	78 %	73 %	73 %	69 %	69 %	61 %	<0.001
Adenosine	21 %	14 %	18 %	20 %	23 %	24 %	<0.001
Dipyridamole		10 %	9 %	9 %	8 %	5 %	0.038
Regadenoson						9 %	–
Dobutamine	1 %	2 %	1 %	1 %	1 %	1 %	n.s.
Adenosine + LLE^a^	^b^	29 %	25 %	22 %	32 %	39 %	n.s.
Dipyridamole + LLE^a^	^b^	57 %	54 %	61 %	64 %	60 %	n.s.
Regadenoson + LLE^a^						51 %	–

Percentages do not always total 100 because of rounding

*LLE* low-level exercise

^a^Percentage of the respective vasodilator stress tests combined with LLE

^b^In 2005, data of adenosine and dipyridamole were not requested

### Gated SPECT and segmental scoring

Gated SPECT is an important adjunct to myocardial perfusion SPECT, but at the beginning of the surveys it showed only a low penetration and acceptance (Table 7, upper half). Over time, gated SPECT experienced a growing increase.

**Table 7:** MPS acquisition as gated SPECT and centres with segmental scoring

		2005	2006	2007	2008	2009	2012	*p* value
Gated SPECT	At stress	32 %	39 %	43 %	42 %	55 %	73 %	<0.001
	At rest	36 %	42 %	46 %	46 %	55 %	70 %	<0.001
	At stress and rest	14 %	36 %	37 %	37 %	47 %	67 %	<0.001
Centres with perfusion scoring	Always			16 %	22 %	33 %	35 %	<0.001
	Intermediate			23 %	15 %	16 %	23 %	0.011
	Never			61 %	62 %	52 %	41 %	<0.001

Percentages do not always total 100 because of rounding

The percentage of centres performing a regular, an intermediate or no quantification with scores is listed in Table 7 (lower half). This feature was queried for the first time in 2007. In 2012, more than 40 % of all centres did not apply any segmental scoring at all. The percentage of centres with a regular scoring was even lower, but slowly increased.

### MPS referrals

Table 8 shows the referral structure of MPS. Ambulatory care cardiologists represented the major referral group, followed by ambulatory care internists and hospitals. In 2012, the cardiologists’ percentage lay well over 50 %, whereas referrals from internists and of inpatients declined. Referrals from primary care physicians and others remained essentially unchanged over the reporting period.

**Table 8:** MPS referrals

	2005	2006	2007	2008	2009	2012	*p* value
Ambulatory care cardiologists	47 %	49 %	47 %	50 %	49 %	60 %	<0.001
Ambulatory care internists	17 %	19 %	23 %	17 %	23 %	14 %	n.s.
Inpatients	21 %	17 %	15 %	16 %	15 %	13 %	n.s.
Primary care physicians	13 %	13 %	12 %	12 %	11 %	10 %	n.s.
Others	2 %	2 %	3 %	5 %	2 %	3 %	n.s.

### Attenuation correction

AC and the technique used were questioned in 2006 and 2009 (Table 9). Both inquiries revealed a low percentage of MPS studies being performed with AC. Transmission source-based AC was used slightly more frequently than CT-based AC. The latter experienced a mild increase from 2006 to 2009.

**Table 9:** AC and methods

	2006	2009
MPS studies with AC	7 %	4 %
No. of centres with CT-based AC	8	12
No. of centres with transmission sources for AC	18	17
No. of centres with both methods	1	3

### Diabetics, age of patients and indications

In 2007 the percentage of diabetics was surveyed. The proportion of diabetics in MPS studies was found to be 21 %. The age structure of the MPS patients was recorded 1 year later. The data revealed that 15 % were aged < 50 years, 57 % 50–70 years and 28 %  > 70 years. In 2006, about 40 % of MPS addressed the issue “suspected CAD”. No further differentiation of indications occurred.

## Discussion

This paper summarizes six surveys on MPS from 2005 to 2012, gives an overview of changes in recent years and demonstrates the current state of MPS practice in Germany. Furthermore, the data allow an estimation of average patient doses from MPS and changes over time.

It should be noted that the surveys represent average and routine use of MPS over a wide user spectrum and not exclusively high-end users. The representativeness of the survey was evaluated in relation to data submitted by the NASHIP, which reimburses services of statutory outpatient care. In relation to the NASHIP numbers, about 50–55 % of all MPS were covered by each survey. The results of all surveys can be regarded as representative. The derived total annual MPS numbers tend to be slightly overestimated by <10 %. This bias applies to all surveys, but does not affect the inter-survey relationships.

The last European survey (reporting year 2007) was published in 2012 and represented 258 European centres, of which 30 were German centres [17]. With data provided from 278–370 centres, the Germany survey data are more comprehensive. Representing about 20 % of the European population, they may also reflect general European MPS practice and development.

The key issues of all six surveys are:

1. Between 2005 and 2012 the average annual utilization rate was 2,590 MPS patients pmp, which was between a moderate and a moderate-high utilization index according to the subdivision by Vitola et al. [18]. Compared with the European surveys, the annual utilization rate lies in the upper half. Nevertheless, the German and the European utilization numbers are in stark contrast to the USA with nearly 30,000 MPS pmp and an annual study number per centre of 1,225 [8, 19]. The reasons are surely to be found in the clear interdisciplinary division between cardiology and nuclear medicine in Germany and the higher reimbursement in the USA. Only three ambulatory care cardiologists practice nuclear cardiology imaging themselves in Germany. Interestingly, these are high utilization centres with annual MPS numbers of >1,500.
2. CAD imaging and decision-making in Germany is clearly morphology driven. The cardiology statistics reveal a high angiography pmp rate which is 4 times higher on average than the MPS pmp rate, and clearly above the European angiography to MPS ratio of 2.5 [17]. A stable angiography to revascularization ratio of 2.3 indicates that far more than 50 % of all angiographies remain without any interventional procedure. The estimate is in line with other overviews which report a normal rate of 20–40 % in angiographies and reveal a suboptimal preselection of patients and obviously no essential change in the past few years despite the results of the COURAGE trial [20, 21].
3. Other non-invasive imaging techniques are unlikely to significantly impact the angiography to non-invasive imaging ratio. Numbers of stress echocardiograms in Germany are not available. The same applies to CT angiography and cardiac perfusion MRI, whose numbers are certainly low because they are not yet reimbursed within the statutory health insurance system.
4. The MPS to revascularization ratio decreased from 0.66 in 2005 to 0.55 in 2012 (note: the MPS 2012 numbers were related to cardiology data from 2011). It is lower than the European ratio of 0.9 in 2007. The value of 0.55 reflects the imbalance between functional and morphological imaging and suggests that many interventions are performed without knowledge of myocardial perfusion/viability. The German diagnosis-related group (DRG) system, which reimburses cases and not single diagnostic steps, supports this trend.
5. About 20–25 % of MPS studies were performed in centres with less than 50 MPS/year (<1 MPS/week) and one tenth in sites with more than 1,000 MPS/year (> 4 MPS/day). Over the reporting period there was a slight trend towards a concentration of MPS studies. This is a favourable observation as competence increases with number of studies. The low number of MPS patient centres should be in the special focus of training and education. This was one motivation to establish control panels by the medical chambers in order to monitor quality standards according to guidelines and radiation protection directives [1].
6. Centres participating in all surveys from 2005 to 2012 reported an increase in their MPS numbers of 4 % on average. Only university hospitals experienced a decline in their numbers, indicating a shift towards preclinical diagnosis. It should be noted that the 108 centres in all surveys are not exactly representative since they are likely to represent a selection of more active and motivated centres.
  On the other hand, a mild increase could also be noticed from 2009 to 2012, and more than 50 % of the centres noticed no change or an increase in MPS imaging in this period.
  To summarize, several indicators support the statement of a positive turnaround in MPS imaging.
7. The survey participants regarded MRI, angiography and stress echocardiography as competitive methods. As stated above, the proportion of MRI in myocardial perfusion imaging is estimated to be low because this service is not yet reimbursed. Angiography showed a slight decline for the first time in 2011. Together with the increase in referrals from cardiologists, this may be another indicator towards more non-invasive imaging. Utilization numbers for the coming years will further clarify the developments and evaluate the statement.

8. Procedural and technical issues
  8.1. ^99m^Tc radiopharmaceuticals (sestamibi and tetrofosmin) are the dominating agents in MPS, with a growing percentage over time. Rest-only studies are very rare, indicating that viability imaging plays a minor role in MPS. The percentage of stress-only protocols is nearly constant and higher than the proportion of 3 % in the USA in 2008 [19]. As about 40 % of MPS are related to patients with suspected CAD (2006 survey data), a higher amount of stress-only studies could be expected. In the setting of a normal stress study, an additional rest study is not of additional benefit [22]. Thus, stress-only protocols represent an effective measure to substantially reduce radiation exposure and should be the objective of future training and education.
    The preferred use of 2-day ^99m^Tc MPS protocols, which allow the application of lower activities than with 1-day protocols, is another issue to reduce radiation exposure. The proportion is clearly higher than in the ASNC survey (5.3 %) [9].
^201^Tl MPS studies significantly decreased from 20 % in 2005 to 5 % in 2012. Compared with the ASNC survey (in 2011 15.6 % of protocols with ^201^Tl dual isotope and 0.7 % with ^201^Tl only), the proportion in Germany is lower [9]. It is a clear guideline statement that ^99m^Tc radiopharmaceuticals are preferred, and it is expected that the use of ^201^Tl will further decline [23].
  8.2. The surveys demonstrate a reduction in average patient dose from 2006 to 2012 by changes in protocol use primarily by the decline of ^201^Tl. The shortage of ^99m^Tc from 2008 to 2009 with a temporary shift to more ^201^Tl imaging did not sustainably affect average patient dose, which is clearly below 10 mSv.
    A single-centre analysis in Italy documented an effective dose reduction with ^99m^Tc-sestamibi imaging from 13.7 mSv to 5.7 mSv from 2002 to 2012 by weight adjustment of activities and new camera technology together with dedicated reconstruction algorithms [24]. Our survey dose data refer to average protocol use in a range of prescribed DRL. They show a similar trend but as to be expected a milder decline as they are not related to a single centre.
    The contribution of AC systems (CT based or with transmission sources) is unlikely to affect our dose data considerably as such systems are still rare and of low additional dose [24].
    To summarize, dose reduction is an ongoing process in myocardial perfusion imaging; effective steps are giving up ^201^Tl, more stress-only studies, 2-day studies instead of 1-day studies and new technology.
  8.3. Dynamic exercise stress is still the preferred form of stress, but decreasing over the reporting period. Pharmacological stress testing has increased, as well as the combination of vasodilator stress and low-level exercise. Regadenoson, available in Germany since September 2011, already accounted for 8 % (ASNC survey 32.6 %) of stress tests in 2012 [8]. A further increase is to be expected.
    One reason for the growing share of vasodilator studies is the aging patient population with co-morbidities making them unable to exercise adequately. The 2008 data revealed that almost 30 % of patients were aged >70 years and 21 % were diabetics (2007 data).
  8.4. Gated SPECT experienced growing acceptance. In 2012 the greatest increase could be documented. Starting with a proportion of only 35 % in 2005, this constitutes favourable progress. A further increase should be achievable considering that 95 % of all studies are performed with ^99m^Tc radiopharmaceuticals and thus potential gated SPECT studies. ECG gating is not a reimbursed service in Germany. This issue certainly contributes to the still incomplete penetration.
  8.5. All German surveys revealed an underuse of segmental scoring of perfusion defects, with only slowly growing acceptance. Guidelines clearly recommend scoring of myocardial perfusion SPECT to meet the full power of MPS for risk stratification and therapeutic management [23, 25]. Thus, perfusion scoring remains a target issue of training and education in nuclear cardiology.
  8.6. Until 2009, AC failed to become a standard in MPS (proportion of less than 10 %). With a broader dissemination of hybrid SPECT/CT cameras, a mild increase is likely.

### Conclusion

The six German surveys on MPS from 2005 to 2012 reflect the competitive context of MPS in the field of mainly morphology-based cardiac imaging. Nevertheless, the data reveal a positive trend in MPS parallelled with a decrease in average patient dose. Both issues and the procedural survey results reflect good routine practice with adherence to the current guidelines, the implementation of technical improvements and success in training over the years. The German experience of the past years may serve as a model for future considerations in other countries.
